# Serum IGF-1 and IGFBP-3 Levels in Healthy Children Between 0 and 6 Years of Age

**DOI:** 10.4274/jcrpe.v3i2.17

**Published:** 2011-06-08

**Authors:** Bilgin Yüksel, M. Nuri Özbek, Neslihan Önenli Mungan, Feyza Darendeliler, Bahar Budan, Aysun Bideci, Ergün Çetinkaya, Merih Berberoğlu, Olcay Evliyaoğlu, Ediz Yeşilkaya, İlknur Arslanoğlu, Şükran Darcan, Ruveyda Bundak, Olcay Ercan

**Affiliations:** 1 Cukurova University, Faculty of Medicine, Pediatric Endocrinology and Metabolism, Adana, Turkey; 2 Diyarbakir Children's Hospital, Pediatric Endocrinology, Diyarbakir, Turkey; 3 Istanbul University, Faculty of Medicine, Pediatric Endocrinology, Istanbul, Turkey; 4 Gazi University, Faculty of Medicine, Pediatric Endocrinology , Ankara, Turkey; 5 Diskapi Hospital, Pediatric Endocrinology , Ankara, Turkey; 6 Ankara University, Faculty of Medicine, Pediatric Endocrinology, Ankara, Turkey; 7 Kirikkale University, Faculty of Medicine, Pediatric Endocrinology, Kirikkale, Turkey; 8 Kirikkale GATA Hospital, Pediatric Endocrinology, Ankara, Turkey; 9 Duzce University, Faculty of Medicine, Pediatric Endocrinology, Duzce, Turkey; 10 Ege University, Faculty of Medicine, Pediatric Endocrinology and Metabolism, Izmir, Turkey; 11 Istanbul University, Cerrahpasa Medical Faculty, Pediatric Endocrinology, Istanbul, Turkey

**Keywords:** childhood, IGF-1, IGFBP-3, Growth hormone deficiency

## Abstract

**Objective:** Along with growth hormone (GH) levels, measurements of serum insulin-like growth factor-1 (IGF-1) and IGF-binding protein-3 (IGFBP-3) are used in the diagnosis of GH deficiency and in monitoring the efficacy and safety of long-term GH treatment. The purpose of the present study was to establish reference values for serum IGF-1 and IGFBP-3 in healthy Turkish children less than 6 years of age.

**Methods:** This study was designed as a multicenter project. Five hundred sixty-seven healthy children younger than 6 years of age from different geographical regions of Turkey, with weight and height values between the 10th and 90th percentiles according to the national standards were included in the study. In addition to anthropometric parameters, serum IGF-1 and IGFBP-3 levels were measured in all subjects.

Results: Although not statistically significant, the serum IGF-1 levels in infants at age 6 months were lower than those in infants at age 3 months. The IGF-1 levels showed a slow increase with age. Serum IGF-1 levels were lower in girls as compared to boys only at age 6 months. No correlation was found between either serum IGFBP-3 levels and body mass index (BMI) or serum IGFBP-3 and weight and height standard deviation scores (SDS). A weak correlation was observed between serum IGF-1 and IGFBP-3 concentrations.

**Conclusions:** The age- and gender-specific reference values for serum IGF-1 and IGFBP-3 reported in this study will aid in the diagnosis of GH deficiency and in the monitoring of children receiving GH treatment.

**Conflict of interest:**None declared.

## INTRODUCTION

Insulin-like growth factor-1 (IGF-1) is an effector hormone which is essential for normal growth in humans and has an important role in mediating the effects of growth hormone (GH) (1,2,3). In the circulatory system, IGF-1 forms a terenary complex with IGF-binding protein-3 (IGFBP-3) and the acid-labile subunit (4,5). This complex serves as a circulatory reservoir for IGF-1. 

Several studies have shown that the serum levels of IGF-1 and IGFBP-3 are GH-dependent (6). Serum IGF-1 and IGFBP-3 concentrations are decreased in patients with GH deficiency and increased in patients with acromegaly (1,2,3,7,8,9). GH is secreted in a pulsatile pattern. On the other hand, serum IGF-1 and IGFBP-3 have almost no pulsatile secretion and for that reason they are used widely in clinics (1,2,7,10,11,12,13,14,15).

Although IGF-1 and IGFBP-3 are mainly secreted by the liver, they may be produced in several other tissues as well. Serum IGF-1 levels increase as the child grows, reach a peak value at puberty, and decrease with aging. Serum IGFBP-3 levels show a relatively similar pattern (1,2,3,16,17,18,19,20,21,22,23,24, 25,26,27). Although GH is the main regulator of the production of IGF-1 and IGFBP-3, other factors, such as gender, puberty, hormones, nutrition, seasonal variations, liver and renal functions, gene polymorphisms also have an effect on their levels (1,2,3,28). Therefore, these confounding factors should be considered when evaluating the serum IGF-1 and IGFBP-3 concentrations. It will also be helpful to have population-specific reference ranges for serum IGF-1 and IGFBP-3 in this evaluation. 

To our knowledge, there is a lack of large-scale studies analyzing the serum IGF-1 and IGFBP-3 levels young children. The purpose of this study was to determine the normal reference ranges of serum IGF-1 and IGFBP-3 in healthy infants and children younger than 6 years of age. 

## MATERIALS AND METHODS

This multicenter study included randomly selected healthy infants and children (310 boys and 256 girls) younger than 6 years of age from different geographical regions of Turkey, with weight and height measurements between the 10th and 90th percentiles by the national standards (29,30). Infants and children who had shown signs or symptoms of infection in the preceding week were excluded. The subjects were categorized into 8 groups according to their chronological age (Table 1). 

Measurements of height, weight, and body mass index (BMI; kg/m2) were expressed as mean±standard deviation (SD) values and calculated according to the national standards (29,30,31).

Morning blood samples (2-3 mL) were obtained from all subjects for IGF-1 and IGFBP-3 measurements. Samples were separated by centrifugation and stored at -20°C until analysis. All samples were studied at the same time.

Serum IGF-1 and serum IGFBP-3 levels were measured with commercially available enzyme-linked immunosorbent assay (ELISA) kits [Diagnostic Systems Laboratories Inc. (DSL) DSL-10-2800 IGF-1 (Active® U.S.A)] and DSL-10-6600 IGFBP-3 (Active® U.S.A), respectively, in accordance with the manufacturer's recommendations. IGF-1 and IGFBP-3 values were expressed as ng/dL. The lower limit of detection was 0.01 ng/mL for IGF-1 and 0.04 ng/mL for IGFBP-3. The intra- and inter-assay coefficients of variation (CV) for IGF-1 were 6.3% and 3.3%, respectively. The intra- and inter-assay CV were 9.6% and 11.4% for IGFBP-3, in the same order. 

Approval was obtained from the Cukurova University Ethics Committee. Families gave informed consent for their child’s participation in the research. This study was conducted in accordance with the Helsinki II Declaration.

The statistical analyses were performed using SPSS (version 16; SPSS, Inc., Chicago, IL, USA). The data were given as SD, mean, minimum and maximum values, and 95% confidence intervals (CI). The Pearson’s correlation coefficient (r) was used to evaluate the relationship of IGF-1 and IGFBP-3 with other variables, while the Spearman’s rank correlation coefficient was used when needed. A p-value of less than 0.05 was considered to be statistically significant.

**Table 1:**
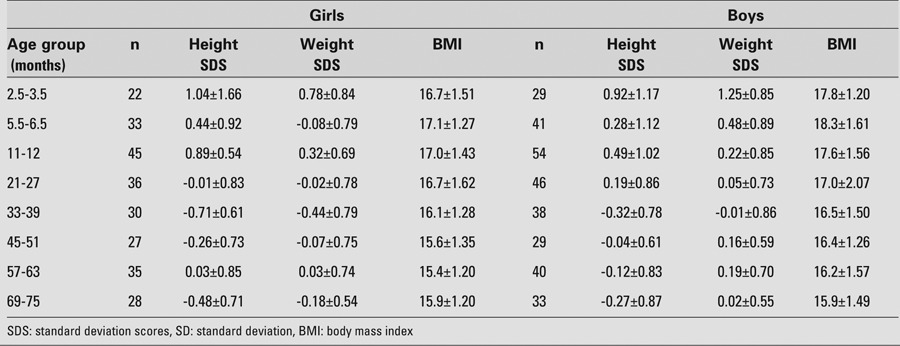
Height SDS, weight SDS, and BMI of the subjects (mean±SD values)

## RESULTS

**Figures 1:**
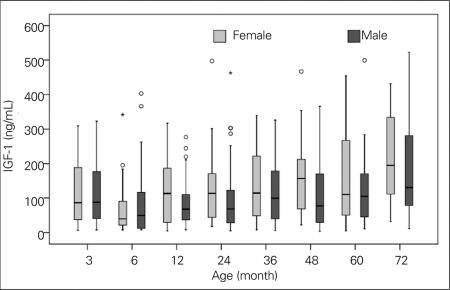
Age distribution of serum IGF-1 levels (ng/mL) in both genders IGF-1: insulin-like growth factor-1

Height SDS, weight SDS, and BMI values of all children were within normal ranges, as shown in Table 1. Mean and SDS values, ranges, and 95% CIs for serum IGF-1 and IGFBP-3 levels in both genders are shown in Table 2. Figures 1 and 2 illustrate the IGF-1 and IGFBP-3 values at different ages. 

The serum IGF-1 levels of infants in the 5.5-6.5-month age group were found to be lower than those of infants in the 2.5-3.5-month group (Table 2, Figure 1). 

IGF-1 level correlated with BMI in girls in the 33-39-month age group and in boys in the age groups 21-27 and 45-51 months (r= 0.564, p= 0.012; r= 0.413, p= 0.023; and r= 0.568, p= 0.009, respectively). By the Spearman’s method, the above correlation was only observed in the 33-39-month age group in girls and in the 45-51-month age group in boys. No association was found between serum IGF-1 levels and BMI at other ages by either method. By the Spearman’s method, a relationship between serum IGF-1 and height SDS was detected only in boys in the age group 57-63 months (r= 0.462, p= 0.01).

A decreased serum IGFBP-3 concentration was only observed in girls at 6 months of age (Table 2, Figure 2). In contrast to IGF-1, serum IGFBP-3 levels did not increase in parallel to the growth of children. 

In the girls, no significant differences were detected in serum IGF-1 levels among age groups. On the other hand, the girls in all age groups, except the 5.5-6.5-month group, had higher IGF-1 values than the boys in all age groups. There was no such relationship for IGFBP-3 levels (Table 2).

No correlations were found between either IGFBP-3 and BMI or IGFBP-3 and height and weight SDS. A weak correlation was observed between serum IGF-1 and IGFBP-3 in some age groups.

**2:**
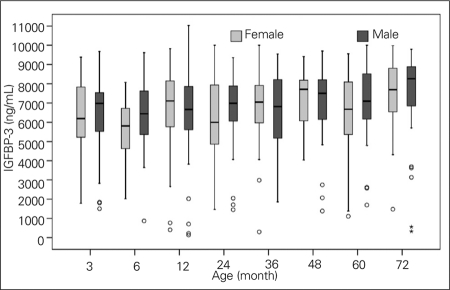
Age distribution of serum IGFBP-3 levels (ng/mL) in both genders IGFBP-3: insulin-like growth factor binding protein-3

**Table 1:**
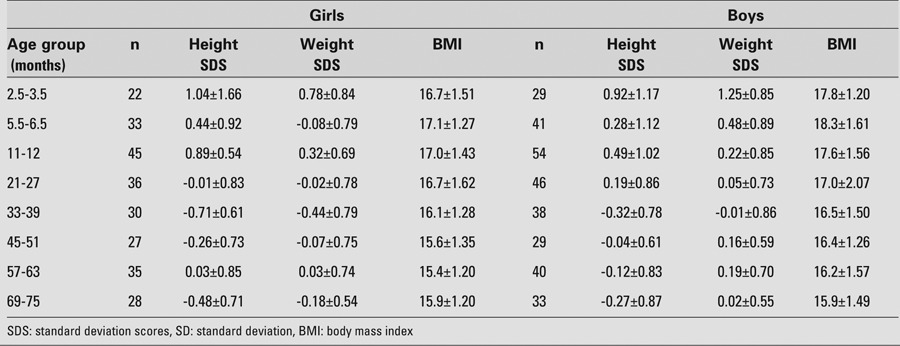
Height SDS, weight SDS, and BMI of the subjects (mean±SD values)

**Table 2:**
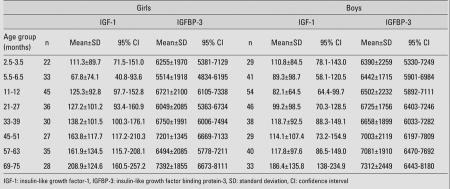
Serum levels (ng/dL) and 95% CI values of IGF-1 and IGFBP-3 of the subjects (mean±SD)

## DISCUSSION

In this study, we established the reference intervals for serum IGF-1 and IGFBP-3 levels in Turkish children younger than 6 years of age. It has been reported that the serum IGF-1 levels tend to increase slowly with age in childhood, reach a peak level at puberty, and decrease with age thereafter. Serum IGF-1 concentrations in female children and adolescents are slightly higher than those in their male counterparts. Additionally, the time to reach a peak value is 2 years earlier in females (3,16,17,18,19,20,24,25). All of these studies established the reference ranges for school-aged children and adolescents and, most of the studies included relatively small numbers of young children under 6 years of age. In agreement with previous reports, our findings also indicate that serum IGF-1 levels tend to increase slowly with age. One of the largest studies which established the reference levels for IGF-1 and IGFBP-3 was conducted on 353 children between 0 and 5 years of age and showed that serum IGF-1 levels decreased slowly between 0 and 24 months but increased with age thereafter (27). Although the decrease found during the first 24 months was not significant, the increase after 24 months was statistically significant (27). We observed a similar non-significant decrease in IGF-1 values in the first 6 months in the girls and in the first 12 months in the boys and an increase thereafter. In a limited number of subjects, Bereket et al (25) reported that serum IGF-1 levels increased slowly at ages 4 to 6 years in healthy children. The ranges of serum IGF-1 and IGFBP-3 concentrations at 4-6 years of age obtained in this study were similar to our results.

Cord blood levels of IGF-1 and IGFBP-3 correlate with gestational age, birth weight, and birth height. However, IGF-1 and IGFBP-3 are not under the influence of GH during the intrauterine period (32). In a prospective study, Chellakooty et al (33) reported that a statistically significant but weak association existed between serum IGF-1 and IGFBP-3 levels and postnatal growth in infants at age 3 months who were appropriate for gestational age (AGA) but not in those who were small for gestational age (SGA). In the same study, serum IGF-1 and IGFBP-3 levels in breast-fed infants were significantly lower than the levels in formula-fed ones (33). In our study, all subjects 6 months of age or less were either exclusively breast-fed or received supplementary food in addition to breastfeeding. The low serum IGF-1 and IGFBP-3 concentrations in the first 6 to 12 months of age might be due to breastfeeding during these months. It has been shown that GH, IGF-1, and IGFBP-3 are present in mother’s milk, and the levels increase with GH treatment (34,35). It may be that the hormones in mother’s milk suppress the GH-IGF axis of the infant. Although most of the studies reported negative correlations between long-term breastfeeding and body weight, there are also studies showing a positive correlation (36). 

Serum IGFBP-3 levels correlate with spontaneous nocturnal GH secretion (10). While normal concentrations of serum IGF-1 and IGFBP-3 rule out the diagnosis of GH deficiency, lower levels support it (11,12). Similar to serum IGF-1 levels, the levels of serum IGFBP-3 also increase with age, reach a peak at puberty, though not as high as serum IGF-1, then decrease with increasing age (7,18). We did not detect any significant elevation in serum IGFBP-3 levels in infants and children younger than 6 years. Males had slightly higher values but not at a statistically significant level. In contrast, Chellakooty et al (33) who measured the serum IGFBP-3 levels 3-month-old infants (with normal birth weights), found significantly higher values in the females. Higher concentrations of serum IGFBP-3 in female children have been noted in other studies as well (7,21,22,25). The gender disparity in IGFBP-3 levels is more prominent at puberty but less pronounced than that for serum IGF-1 levels. 

Serum IGF-1 levels were shown to have a positive correlation with BMI. However, a precise association between BMI and serum IGFBP-3 was not established (21,25). The positive correlation between serum IGF-1 and IGFBP-3 levels and BMI is most prominent in puberty (r,^25^,^27^). In our study, we found a weak correlation between BMI and serum IGF-1 levels in infants and young children. However, serum IGF-1 was found to strongly correlate with BMI in other studies, though mostly conducted among school-aged children and adolescents. This finding suggests that the correlation becomes stronger with age and pubertal development. In our study, no association was observed between IGFBP-3 and BMI in any age group.Serum IGF-1 and serum IGFBP-3 levels are known to positively correlate with normal growth rate in older children and adolescents and also with growth rate in children receiving GH therapy (7,14,15). It is not always possible to show this relationship in cross-sectional studies, such as ours. Further prospective studies are required to demonstrate whether this correlation also exists in prepubertal children. In conclusion, this study served to establish the reference levels for serum IGF-1 and IGFBP-3 in healthy Turkish infants and children below 6 years of age. These values can be used as reference values in screening for GH deficiency and also in monitoring GH treatment for efficacy and safety in children with GH deficiency.
